# Spanish Validation of the Emotional Reactions to Challenging Behaviours Scale in Employees Working with People Exhibiting Intellectual Disabilities

**DOI:** 10.3390/ijerph19010219

**Published:** 2021-12-25

**Authors:** Pilar Montañés Muro, Francisco Lucas Chinchilla Hernández, Guadalupe Manzano García, Renzo Bianchi

**Affiliations:** 1 Deparment of Sciencies Education, Faculty of Law and Social Sciences, University of La Rioja, 26500 Logroño, Spain; francisco.chinchilla@unirioja.es (F.L.C.H.); guadalupe.manzano@unirioja.es (G.M.G.); 2 Institute of Work and Organizational Psychology, University of Neuchâtel, 2000 Neuchâtel, Switzerland; renzo.bianchi@unine.ch

**Keywords:** emotional reactions to challenging behaviours scale, reliability, validity, confirmatory factor analysis

## Abstract

The Emotional Reactions to Challenging Behaviours Scale (ERCBS) was designed to evaluate the emotional states of support staff facing challenging behaviours from recipients. Employees working with People Exhibiting Intellectual Disability (PEIDs) are exposed to challenging behaviours. Exposure to challenging behaviours can affect the occupational well-being of these employees. It is thus important for investigators to have instruments assessing employees’ emotional reactions to challenging behaviours reliably and validly. In this study, we translated the ERCBS into Spanish and inquired into the psychometric and structural properties of the adapted instrument. We relied on a sample of 232 employees working with PEIDs. Confirmatory factor analysis indicated that the ERCBS was best modelled as a two-factor measure involving a negative emotion factor and a positive emotion factor. The two factors were highly correlated (0.547), suggesting that a distinction between “emotional” and “non-emotional” individuals might be relevant when using the ERCBS. Alpha and omega reliabilities were satisfactory. ERCBS scores did not differ between men and women. ERCBS scores correlated negatively with participants’ age and years of service with PEIDs. Our study suggests that the Spanish version of the ERCBS can be used to assess emotional reactions to challenging behaviours among employees working with PEIDs.

## 1. Introduction

Intellectual Disability (ID) is defined as “an individual condition or state characterized by significant limitations in intellectual functioning and in conceptual, social, and practical adaptive skills. This disability originates during the developmental period” [1]. People with ID are more vulnerable to mental health, physical health, and behavioural problems [2,3].

Emerson [4] (p. 3) defined challenging behaviour(s) as “culturally abnormal behaviour(s) of such an intensity, frequency or duration that the physical safety of the person or others is likely to be placed in serious jeopardy, or behaviour which is likely to seriously limit use of, or result in the person being denied access to, ordinary community facilities”. Challenging behaviours notably include aggression, self-injury, destructiveness, overactivity, inappropriate social or sexual conduct, and bizarre mannerisms. Addressing and understanding these behaviours can be difficult and stressful for support staff and family members. As noted earlier, these behaviours can threaten the integrity of individuals with ID and of those who surround them. In most cases, challenging behaviour is a way of communicating and expressing difficulties, needs, fears, or desires [5,6].

It is estimated that 30–60% of people with ID have some form of behavioural disorder [4]. There is evidence that individuals with ID are three to five time more likely to produce challenging behaviours compared to their typically-developing counterparts [7,8]. Behavioural problems negatively affect the health, well-being, and quality of life of people with ID. As pointed out by Emerson [4], the personal and social consequences of these behavioural problems can be immediate (e.g., damage to the person’s health and quality of life) or long-term (e.g., abuse, neglect, inappropriate treatment, exclusion, or social deprivation). The behavioural problems most frequently observed in people with ID include [9]: (a) behaviours that put their health and physical integrity at risk (e.g., self-harm, escape behaviours, refusing to eat or sleep, ingesting inedible substances); (b) behaviours that pose a safety risk to people supporting them or surrounding them (e.g., physical aggression), in addition to hetero-aggressive behaviours intended to destroy materials in the physical environment; (c) oppositional behaviours or persistent refusal of caregivers’ demands (e.g., disobedience, immobility, mutism); (d) disruptive or annoying behaviours that challenge the helper (such as persistent yelling, insolence, abnormal sleep patterns, motor overactivity) or break social norms (e.g., regurgitating food, handling faeces), and (e) emotional regulation problems that manifest themselves as negative emotional disturbances with extreme irritability or uncontrolled emotions. These behaviours sometimes appear when faced with a highly positive emotion that the subject is unable to reduce in intensity (hyper-excitability).

Individuals with ID need support to function in their environment. In most community environments, people with ID encounter barriers to involvement and participation, notably because of a lack of means to promote cognitive accessibility [10]. Direct Care Workers (DCWs) intervene directly to help people with ID integrate into their community and environment.

In addition, DCWs play an important role at the level of emotional regulation. People with ID often have difficulties identifying, expressing, and coping adequately with negative and positive emotional states in their daily lives. DCWs help individuals with ID regulate their emotions and behaviours in order to reduce the risk of negative outcomes. All in all, it is thus common for these workers to be exposed to the challenging behaviours produced by people with ID.

The relationship between exposure to challenging behaviours and the occupational well-being of staff working with people exhibiting ID has been widely investigated [11,12,13]. Several studies have identified a link between exposure to challenging behaviours (notably, aggressiveness and physical and verbal violence) and increased levels of stress and fatigue among DCWs [14,15,16,17]. Exposure to patients’ challenging behaviours has been linked to increased personal anxiety [18], burnout symptoms [11,19,20,21,22,23], and negative physical and emotional responses by staff [24].

In light of this state of affairs, it is important to develop measures of staff’s emotional reactions to the challenging behaviours produced by people with ID. Such instruments can enable us to intervene and provide support staff with strategies to deal with challenging behaviours. In Spain, one of the most widely used instruments for assessing emotions is the Positive and Negative Affect Scale (PANAS) [25]; see also [26,27,28]. The PANAS focuses on people’s general emotional states. In contrast, the Emotional Reactions to Challenging Behaviours Scale (ERCBS) [29,30] specifically evaluates the emotional states of support staff encountering challenging behaviours. The ERCBS was originally developed by Mitchell and Hastings [29] to evaluate staff’s negative emotions in the face of challenging user behaviours. Later, Jones and Hastings [30] included evaluation of positive emotions. The ERCBS consists of four subscales, of which two assess positive emotions (cheerfulness/excitement and confident/relaxed) and two assess negative emotions (depression/anger and fear/anxiety). Until now, the ERCBS has been validated only in a limited number of countries and languages [31,32,33]. In Europe, Zijlmans et al. [31] translated the ERCBS into Dutch. These authors reported Cronbach’s alphas ranging from 0.69 to 0.79 across the four subscales of the measure. Willems et al. [32] used the same scale but grouped the items into a positive emotion subscale and a negative emotion subscale, obtaining Cronbach’s alphas of 0.84 and 0.82, respectively. In Asia, Oh, Seo and Kozub [33] created a modified Korean version of the ERCBS. These authors extracted five subscales, with Cronbach’s alphas ranging from 0.71 to 0.87. Experiencing high negative emotional reactions associated with prolonged exposure to challenging behaviours can be a precedent to job stress and burnout of these professionals [34,35]. Therefore, having a tool that allows us to detect negative emotional states of staff would help those service manager to create more specific support and intervention programs for staff who are in this situation of risk and mitigate the impact of challenging behaviours [36].

In this study, we translated the ERCBS into Spanish and inquired into the psychometric and structural properties of the adapted instrument. We relied on a sample of support staff members working with people exhibiting ID. We specifically examined (a) the factorial structure and reliability of the ERCBS, and (b) the phenotypic expression of ERCBS scores across sexes and age groups.

## 2. Materials and Methods

### 2.1. Participants

A total of 232 DCWs for people with ID were recruited in different centres in Spain, specifically in the regions of La Rioja, Navarra, and Castilla-la Mancha. Participants were recruited by accidental sampling. Participants’ age ranged from 19 to 62 (*M* = 38.96, *SD* = 11.19). Most participants were women (84%). About 45% of the participants were working in day centres and the remaining 55% in care homes. About 4% of the participants were working in the early attention service (users aged 0 to 6), 11% in the junior–adolescent service (users aged 7 to 17), and 85% in adult service (users aged 18 or over). The average number of years participants had been working with the collective was 11.19 (ranging from 1 to 32). With regard to the type of contract, 70% had a permanent contract and 30%, a temporary contract. 81% were working full-time and 19%, part-time.

### 2.2. Instruments

Our measure of interest was the ERCBS [29,30]. The instrument comprises 23 items, divided into four subscales: confident/relaxed (e.g., self-assured), cheerful/excited (e.g., invigorated), fear/anxiety (e.g., afraid) and depression/anger (e.g., guilty). The response categories are 0 (“no, never”), 1 (“yes, sometimes”), 2 (“yes, frequently”), and 3 (“yes, very frequently”). Respondents are asked to indicate the extent to which they experienced specific emotions when confronted with, or dealing with, challenging behaviours.

For adapting the ERCBS, we followed the guidelines of the Second Edition of the International Test Commission [37]. The questionnaire was translated in three stages. In the first stage, two independent translators made an initial translation of the ERCBS into Spanish. Each translator produced a separate translation. The two translators then met up to discuss the overlaps and discrepancies between the two translations and agreed on a common version. In the second stage, the unified translation was reviewed by two experts in clinical psychology and three university professors with high proficiency in English. After reviewing the scores and comments made by each expert, no corrections or modifications to the unified translation were judged necessary. In the third and final stage of the process, we searched for bilingual teachers specialised in translating texts from English into Spanish and vice versa to translate the preliminary version of the questionnaire back into English (back-translation process). The match between the original and the back-translated version was analysed. Both documents were sent to the original author of the scale, Richard Hastings. The author sent us an e-mail approving the Spanish version of the ERCBS.

### 2.3. Procedure

A letter presenting the project was sent out to different Spanish associations that provide support to people with ID to request participation. Once the directors and managers agreed to participate, a Spanish version of the questionnaire was created using the Qualtrics program and distributed. Individual consent to participate was requested. Each association was responsible for distributing the questionnaire to the DCWs working with people with ID, inviting them to participate freely in the study. Participants agreed to participate in the study. The ethics committee of the University of La Rioja (EC-14-2021) approved this study.

### 2.4. Data Analyses

We first computed basic descriptive statistics for the ERCBS, including inter-item correlations. Second, we examined the factorial structure of the ERCBS within a theory-driven framework, relying on confirmatory factor analysis. Based on past research on the ERCBS [29,30], we examined a four-factor model. The five fear–anxiety items were allowed to load on a fear–anxiety factor; the ten depression-anger items were allowed to load on a depression–anger factor; the four confident-relaxed items were allowed to load on a confident–relaxed factor; the four cheerful-excited items were allowed to load on a cheerful–excited factor. We treated the items as ordinal and used the weighted least squares—mean and variance adjusted—estimator. We relied on the following fit indices (see Kline [38]): The Root Mean Square Error of Approximation (RMSEA), the Comparative Fit Index (CFI), the Tucker–Lewis Index (TLI), and the Standardized Root Mean Squared Residual (SRMR). We evaluated the fit indices based on commonly accepted cut-points. The cut-points were 0.08 for the RMSEA, 0.95 for the CFI and TLI, and 0.08 for the SRMR. Third, the reliability of the ERCBS was estimated using McDonald’s omega and Cronbach’s alpha. Fourth, we examined whether ERCBS total scores and subscale scores differed across sexes and services using analysis of variance (ANOVA), and we looked into the correlations of ERCBS total scores and subscale scores with age and years of service. We conducted our analyses in Mplus 8.6 (Muthén & Muthén, Los Angeles, USA) and SPSS (IBM Corp, Armonk, New York, NY, USA).

## 3. Results

### 3.1. Descriptive Analysis

Descriptive statistics for the ERCBS are available in Table 1. Pearson and Spearman correlations of the ERCBS items with the ERCBS total score varied from 0.43 to 0.78 (all *p* < 0.001). Inter-item correlations varied from small to large (Table 2).

### 3.2. Factorial Structure

**Figure 1:**
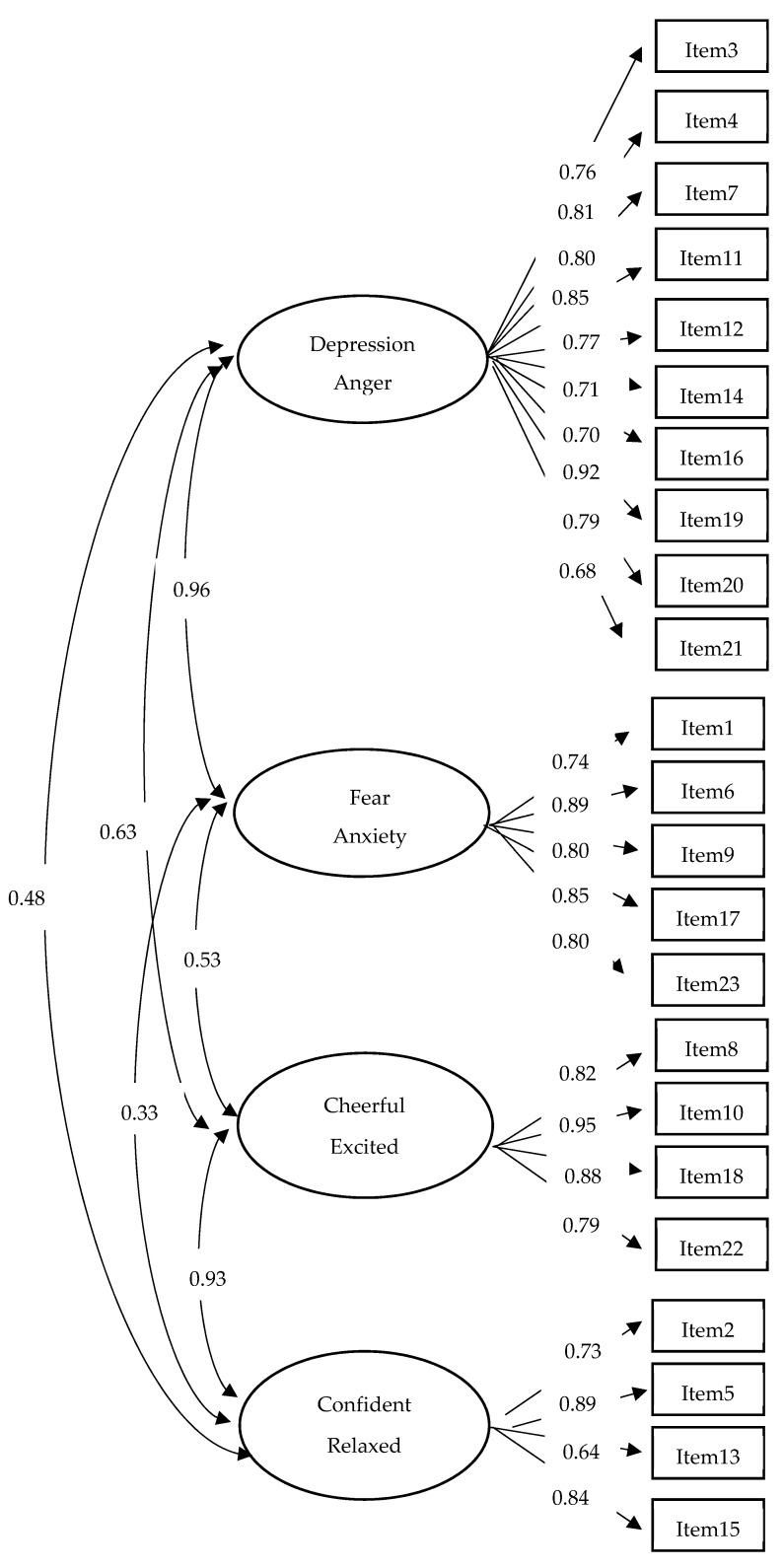
Standardized factor loadings for the four-factor model.

**Figure 2:**
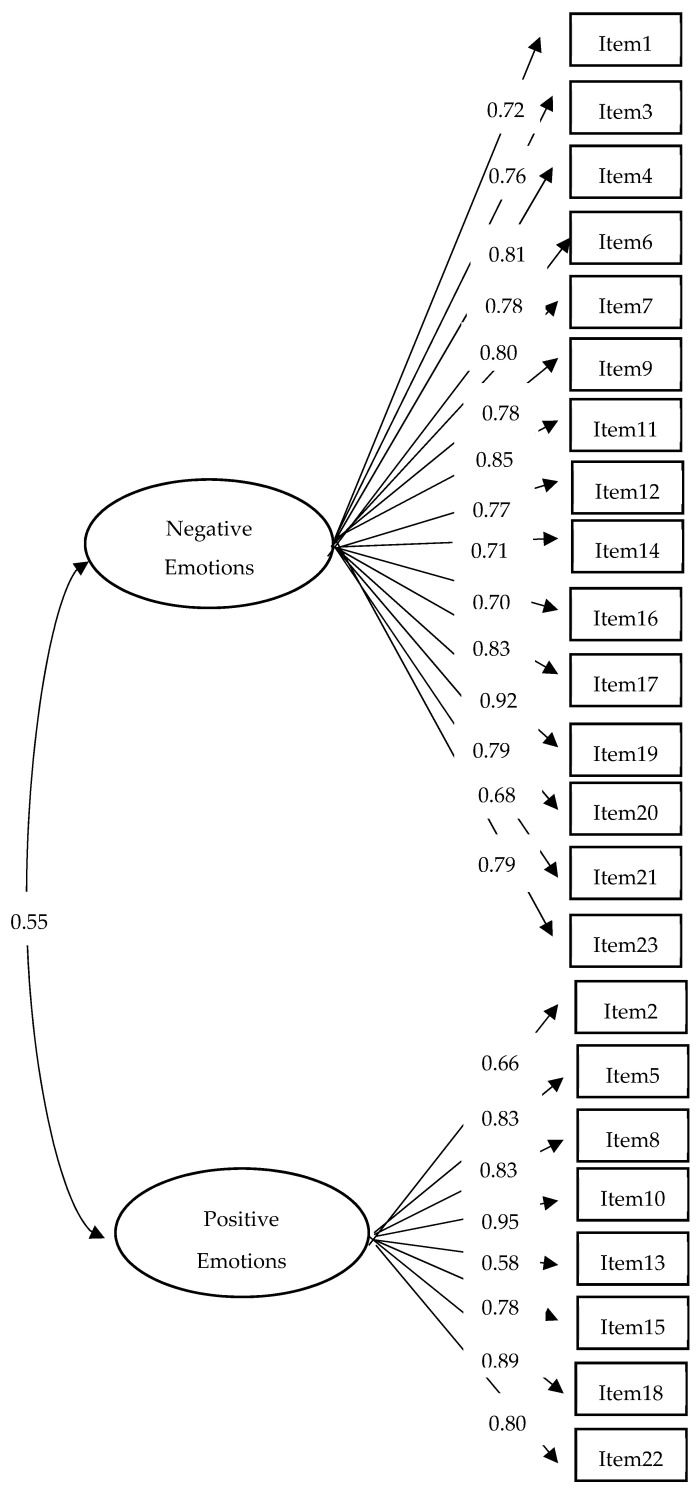
Standardized factor loadings for the two-factor model.

Our four-factor model showed an acceptable fit (Table 3; the standardized factor loadings are displayed in Figure 1). However, close-to-perfect correlations were observed between the fear–anxiety and depression–anger factors on the one hand (0.96), and between the confident–relaxed and cheerful–excited factors on the other hand (0.93). This state of affairs led us to consider a two-factor model in which all negative affectivity items were allowed to load on a negative affectivity factor and all positive affectivity items were allowed to load on a positive affectivity factor. The two-factor model exhibited an acceptable, though slightly degraded, fit (Table 3). The standardized factor loadings for the two-factor model are displayed in Figure 2. The negative affectivity and positive affectivity factors correlated 0.547, suggesting that individuals who endorsed negative affectivity items were also more likely to endorse positive affectivity items. This finding suggests that a distinction between “emotional” and “non-emotional” individuals might be relevant in addition to a distinction based on the valence (negative/positive) of the emotions embodied in the ERCBS items.

### 3.3. Reliability

Cronbach’s alphas for the four hypothetical ERCB subscales were as follows: 0.92 for the depression-anger subscale; 0.86 for the fear-anxiety subscale; 0.88 for the cheerful-excited subscale; and 0.82 for the confident-relaxed subscale. McDonald’s omegas were 0.92 for the depression-anger subscale; 0.87 for the fear-anxiety subscale; 0.89 for the cheerful-excited subscale; and 0.83 for the confident-relaxed subscale. Cronbach’s alphas were 0.95 for a subscale combining negative emotion items (McDonald’s omega = 0.96), and 0.91 for a subscale combining positive emotion items (McDonald’s omega = 0.93)

### 3.4. ERCBS, Sex, Service, and Age

A first ANOVA revealed no statistically significant effect of sex on ERCBS total scores, *F* (1, 231) = 0.903, *p* = 0.34, or on any of the four subscale scores (depression-anger subscale: *F* (1, 231) = 0.314, *p* = 0.58; fear-anxiety subscale: *F* (1, 231) = 0.064, *p* = 0.80; cheerful-excited subscale: *F* (1, 231) = 1.018, *p* = 0.31; confident-relaxed subscale: *F* (1, 231) = 2.104, *p* = 0.15).

A second ANOVA revealed a statistically significant effect of service on ERCB total scores, *F* (1, 231) = 13.219, *p* = 0.000. People working in the adult service had fewer emotional reactions than people working in the junior–adolescent service (*p* = 0.000) or in the early attention service (*p* = 0.046). There was no difference between people working in the junior–adolescent service or in the early attention service. In relation to the subscales of the ERCBS, our ANOVAs indicated a statistically significant effect of service on the four subscales. (depression–anger subscale: *F* (1, 231) = 12.231, *p* = 0.000; fear–anxiety subscale: *F* (1, 231) = 9.738, *p* = 0.000; cheerful–excited subscale: *F* (1, 231) = 7.746, *p* = 0.000; confident–relaxed subscale: *F* (1, 231) = 5.259, *p* = 0.006. On none of the subscales were there any differences between people working in the junior–adolescent service or in the early attention service. Regarding the depression-anger subscale, adult service workers had lower scores than junior–adolescent service workers (*p* = 0.000) and early attention service workers (*p* = 0.003). Regarding the fear-anxiety subscale, adult service workers had lower scores than junior–adolescent service workers (*p* = 0.005) and early attention service workers (*p* = 0.004). Regarding the cheerful-excited subscale, adult service workers had lower scores than junior–adolescent service workers (*p* = 0.000). Regarding the confident-relaxed subscale, adult service workers had lower scores than junior–adolescent service workers (*p* = 0.004).

Pearson and Spearman correlations indicated that participants’ age and years of service with ID patients were negatively associated with ERCBS total scores and with the four subscales (see Table 4).

## 4. Discussion

In this study, we translated the ERCBS into Spanish and inquired into the psychometric and structural properties of the adapted instrument. We relied on a sample of support staff for people with ID. We found the ERCBS to exhibit acceptable factorial validity and reliability. The initial, four-factor model proposed by the creators of the ERCBS showed an acceptable fit in our study, but given the almost perfect (>0.90) correlations among some of the factors, a two-factor solution involving negative emotion and positive emotion factors appeared to be more appropriate. With respect to reliability, McDonald’s omegas and Cronbach’s alphas all exceeded 0.80. These values are higher than those documented in many ERCBS studies [29,30,33,34,39,40,41].

Regarding the validation of the instrument across cultural contexts, we found only one relevant study, conducted in South Korea [33]. The study in question was based on a previous analysis of the original ERCBS, in which substantial cross-loadings were identified among items related to the depression/anger and fear/anxiety subscales of the instrument [42]. In the validation study of the ERCBS in Korea, a suggestion was made to broaden the scale to 29 items for a three-factor model to fit (positive emotional reactions, reactions of depression/anger, and reactions of fear/anxiety). After an Exploratory Factor Analysis (EFA), the 29 items were reduced to 28. A subsequent Confirmatory Factor Analysis (CFA) backed a modified final version of 28 items of the ERCBS-K with a more convincing adjustment index, which endorses a five-factor solution, producing a new five-factor model with a one subscale of positive items and four subscales of negative items (fear/anxiety, responsibility, depression and confusion). After this study a very different three-factor ERCBS of 23 items was obtained by Jones and Hastings [30].

We found no sex differences in ERCBS scores, consistent with the results of several past studies [34,43]. However, in a study involving 83 support staff for people with ID, Mitchell and Hastings [29] found that men (*n* = 38) had higher depression/anger scores than women. Larger-sample studies could help clarify the association of ERCBS scores with sex.

The negative associations of participants’ age and years of service with participants’ ERCBS scores suggest that more experienced individuals are less emotionally reactive to ID people’s challenging behaviours. These results are consistent with previous findings indicating that more experienced workers tend to be less emotionally reactive [39]. Such findings may be helpful in developing training and curricular development programs for staff dealing with ID patients. The Positive Behaviour Support Training (PBS) has proved effective in reducing negative emotional reactions among staff dealing with ID people [40,44]. On a different note, we found that people in the adult service reported fewer emotional reactions than people working in the youth service.

Interestingly, the strong correlation that we observed between the positive emotion factor and the negative emotion factor suggests that the ERCBS distinguishes between “emotional” and “non-emotional” individuals. Support staff who experience more intense negative emotional reactions to the presence of challenging behaviours are more likely to experience greater job stress, emotional exhaustion, depersonalization, and personal accomplishment among disability support workers [16,23]. Therefore, it is important to have an instrument that can help organizations preventively detect these symptoms.

Our study has at least three limitations. First, it would have been preferable to have a larger sample. We note, however, that participant recruitment is challenging when focusing on highly specific occupational groups, as is the case in this study. Second, our study was conducted using a convenience sample coming from a specific region of Spain. Third, we had considerably more participants working in the adult service than in the early attention service or the junior–adolescent service, suggesting that our service-related analyses should be interpreted with caution. Future research should attempt to replicate these results with a broader geographic scope and a sample representative of its population of reference (e.g., in terms of basic sociodemographic characteristics).

This study indicates that the Spanish version for the ERCBS shows acceptable factorial validity and reliability. To our knowledge, this is the first validation of the instrument with a Spanish sample and the only validation that did not modifications in the original content of the instrument. Given the relationship between exposure to challenging behaviours and the occupational well-being [11,12,13], it is important to provide support staff with strategies enabling them to deal more effectively with challenging behaviours. The ERCBS may be useful to develop interventions focused on the specific emotional reactions of support staff for people with ID.

## 5. Conclusions

This study suggests that the Spanish version of the ERCBS can be used to assess emotional reactions to challenging behaviours among employees working with people exhibiting intellectual disability (PEIDs). The ERCBS was best modelled as a two-factor measure involving a negative emotion factor and a positive emotion factor. On the other hand, the age of the participants and their years of service with PEIDs were related to fewer emotional reactions to challenging behaviours from those with ID. Finally, no differences were found in ERCBS scores according to the sex of participants.

## Figures and Tables

**Table 1:** Descriptive statistics for the items (N = 232).

	M	SD	G1	G2
Item 1. Shocked	1.70	0.94	−0.06	−0.55
Item 2. Confident	1.98	1.10	−0.26	−0.72
Item 3. Guilty	1.03	0.83	0.37	−0.58
Item 4. Hopeless	1.52	0.97	0.22	−0.41
Item 5. Comfortable	1.28	1.13	0.64	−0.49
Item 6. Afraid	1.55	0.91	0.06	−0.32
Item 7. Angry	1.38	0.92	0.31	−0.25
Item 8. Invigorated	1.42	1.08	0.21	−0.98
Item 9. Incompetent	1.11	0.89	0.48	−0.12
Item 10. Happy	1.15	1.17	0.88	−0.22
Item 11. Frustrated	1.90	1.05	−0.04	−0.63
Item 12. Helpless	1.61	0.99	0.29	−0.42
Item 13. Self-assured	2.30	0.90	−0.30	−0.05
Item 14. Disgusted	2.01	0.99	−0.11	−0.46
Item 15. Relaxed	1.27	0.98	0.54	−0.31
Item 16. Resigned	1.37	0.97	0.31	−0.48
Item 17. Frightened	1.53	0.90	0.16	−0.11
Item 18. Cheerful	1.38	1.22	0.55	−0.85
Item 19. Humiliated	0.84	0.76	0.70	0.26
Item 20. Betrayed	0.82	0.75	0.81	0.73
Item 21. Sad	1.62	0.99	0.26	−0.33
Item 22. Excited	1.53	1.08	0.16	−0.82
Item 23. Nervous	1.94	0.95	0.15	−0.62

Notes: M = mean; SD = standard deviation; G1 = asymmetry; G2 = Kurtosis.

**Table 2:** Inter-item correlations.

	1	2	3	4	5	6	7	8	9	10	11	12	13	14	15	16	17	18	19	20	21	22	23	ERCB-T
Item 1. Shocked	-	0.237 **	0.546 **	0.647 **	0.291 **	0.615 **	0.531 **	0.346 **	0.483 **	0.459 **	0.573 **	0.498 **	0.259 **	0.507 **	0.294 **	0.407 **	0.504 **	0.401 **	0.599 **	0.550 **	0.477 **	0.425 **	0.538 **	0.696 **
Item 2. Confident	0.237 **	-	0.358 **	0.170 *	0.590 **	0.095	0.232 **	0.567 **	0.241 **	0.516 **	0.170 *	−0.021	0.578 **	0.159 *	0.483 **	0.173 **	0.092	0.532 **	0.285 **	0.301 **	0.086	0.441 **	0.114	0.463 **
Item 3. Guilty	0.508 **	0.342 **	-	0.573 **	0.401 **	0.526 **	0.561 **	0.487 **	0.606 **	0.509 **	0.624 **	0.438 **	0.314 **	0.482 **	0.398 **	0.415 **	0.482 **	0.502 **	0.600 **	0.559 **	0.441 **	0.576 **	0.523 **	0.756 **
Item 4. Hopeless	0.626 **	0.163 *	0.565 **	-	0.293 **	0.614 **	0.618 **	0.373 **	0.553 **	0.444 **	0.679 **	0.571 **	0.207 **	0.534 **	0.275 **	0.501 **	0.581 **	0.346 **	0.644 **	0.592 **	0.586 **	0.440 **	0.575 **	0.752 **
Item 5. Comfortable	0.240 **	0.582 **	0.332 **	0.249 **	-	0.200 **	0.371 **	0.652 **	0.371 **	0.795 **	0.252 **	0.086	0.450 **	0.137 *	0.683 **	0.272 **	0.159 *	0.710 **	0.371 **	0.451 **	0.187 **	0.544 **	0.156 *	0.617 **
Item 6. Afraid	0.585 **	0.075	0.508 **	0.600 **	0.142 *	-	0.614 **	0.250 **	0.539 **	0.354 **	0.681 **	0.697 **	0.100	0.566 **	0.213 **	0.464 **	0.771 **	0.327 **	0.686 **	0.591 **	0.523 **	0.340 **	0.669 **	0.711 **
Item 7. Angry	0.491 **	0.214 **	0.536 **	0.608 **	0.298 **	0.579 **	-	0.335 **	0.489 **	0.447 **	0.655 **	0.515 **	0.176 **	0.537 **	0.292 **	0.551 **	0.528 **	0.360 **	0.703 **	0.676 **	0.503 **	0.433 **	0.583 **	0.740 **
Item 8. Invigorated	0.325 **	0.571 **	0.450 **	0.357 **	.635 **	0.239 **	0.303 **	-	0.377 **	0.716 **	0.312 **	0.091	0.469 **	0.167 *	0.589 **	0.291 **	0.209 **	0.698 **	0.362 **	0.400 **	0.207 **	0.515 **	0.177 **	0.632 **
Item 9. Incompetent	0.461 **	0.225 **	0.558 **	0.531 **	0.310 **	0.498 **	0.457 **	0.325 **	-	0.435 **	0.618 **	0.515 **	0.173 **	0.529 **	0.300 **	0.509 **	0.522 **	0.403 **	0.616 **	0.548 **	0.474 **	0.442 **	0.508 **	0.709 **
Item 10. Happy	0.365 **	0.484 **	0.420 **	0.372 **	0.755 **	0.271 **	0.342 **	0.679 **	0.337 **	-	0.353 **	0.202 **	0.473 **	0.224 **	0.677 **	0.337 **	0.255 **	0.829 **	0.449 **	0.507 **	0.214 **	0.645 **	0.238 **	0.736 **
Item 11. Frustrated	0.550 **	0.161 *	0.618 **	0.667 **	0.201 **	0.654 **	0.639 **	0.307 **	0.595 **	0.261 **	-	0.618 **	0.125	0.627 **	0.200 **	0.475 **	0.628 **	0.275 **	0.687 **	0.571 **	0.611 **	0.412 **	0.676 **	0.742 **
Item 12. Helpless	0.482 **	−0.035	0.406 **	0.567 **	0.032	0.685 **	0.524 **	0.075	0.494 **	0.120	0.607 **	-	0.014	0.520 **	0.109	0.393 **	0.659 **	0.171 **	0.640 **	0.539 **	0.528 **	0.281 **	0.582 **	0.600 **
Item 13. Self-assured	0.232 **	0.573 **	0.283 **	0.173 **	0.450 **	0.080	0.138 *	0.472 **	0.130 *	0.454 **	0.102	0.005	-	0.176 **	0.451 **	0.116	0.061	0.486 **	0.327 **	0.315 **	0.101	0.378 **	0.023	0.432 **
Item 14. Disgusted	0.501 **	0.169 *	0.467 **	0.533 **	0.111	0.549 **	0.549 **	0.151 *	0.506 **	0.135 *	0.606 **	0.512 **	0.167 *	-	0.076	0.327 **	0.549 **	0.133 *	0.565 **	0.537 **	0.634 **	0.267 **	0.641 **	0.615 **
Item 15. Relaxed	0.258 **	0.488 **	0.379 **	0.242 **	0.662 **	0.204 **	0.240 **	0.592 **	0.261 **	0.664 **	0.167 *	0.086	0.454 **	0.056	-	0.262 **	0.075	0.720 **	0.338 **	0.408 **	0.132 *	0.505 **	0.056	0.564 **
Item 16. Resigned	0.396 **	0.173 **	0.408 **	0.489 **	0.266 **	0.459 **	0.543 **	0.280 **	0.480 **	0.298 **	0.491 **	0.393 **	0.115	0.320 **	0.261 **	-	0.400 **	0.300 **	0.507 **	0.510 **	0.351 **	0.324 **	0.479 **	0.610 **
Item 17. Frightened	0.491 **	0.062	0.461 **	0.562 **	0.118	0.763 **	0.526 **	0.197 **	0.495 **	0.181 **	0.626 **	0.673 **	.032	0.550 **	0.069	0.401 **	-	0.140 *	0.645 **	0.581 **	0.568 **	0.292 **	0.683 **	0.640 **
Item 18. CheerfuL	0.341 **	0.509 **	0.431 **	0.297 **	0.679 **	0.278 **	0.279 **	0.677 **	0.329 **	0.818 **	0.217 **	0.117	0.493 **	0.086	0.697 **	0.284 **	0.089	-	0.412 **	0.432 **	0.137 *	0.638 **	0.126	0.675 **
Item 19. Humiliated	0.574 **	0.205 **	0.524 **	0.611 **	0.266 **	0.639 **	0.696 **	0.292 **	0.558 **	0.318 **	0.647 **	0.647 **	0.249 **	0.523 **	0.262 **	0.507 **	0.627 **	0.298 **	-	0.815 **	0.617 **	0.465 **	0.573 **	0.783 **
Item 20. Betrayed	0.494 **	0.198 **	0.467 **	0.536 **	0.340 **	0.553 **	0.631 **	0.309 **	0.487 **	0.350 **	0.527 **	0.534 **	0.230 **	0.509 **	0.311 **	0.491 **	0.565 **	0.302 **	0.783 **	-	0.588 **	0.483 **	0.526 **	0.762 **
Item 21. Sad	0.493 **	0.072	0.425 **	0.575 **	0.147 *	0.490 **	0.492 **	0.193 **	0.436 **	0.130	0.605 **	0.534 **	0.077	0.634 **	0.108	0.333 **	0.568 **	0.097	0.575 **	0.551 **	-	0.357 **	0.546 **	0.610 **
Item 22. Excited	0.412 **	0.441 **	0.564 **	0.429 **	0.507 **	0.330 **	0.421 **	0.510 **	0.419 **	0.605 **	0.417 **	0.290 **	0.374 **	0.282 **	0.489 **	0.326 **	0.305 **	0.614 **	0.429 **	0.427 **	0.360 **	-	0.278 **	0.686 **
Item 23. Nervous	0.511 **	0.091	0.507 **	0.562 **	0.105	0.645 **	0.594 **	0.164 *	0.475 **	0.131 *	0.668 **	0.582 **	0.009	0.645 **	0.033	0.479 **	0.680 **	0.055	0.549 **	0.506 **	0.548 **	0.273 **	-	0.618 **
ERCB-T	0.697 **	0.492 **	0.741 **	0.746 **	0.592 **	0.705 **	0.729 **	0.634 **	0.684 **	0.662 **	0.744 **	0.600 **	0.434 **	0.622 **	0.555 **	0.608 **	0.648 **	0.631 **	0.765 **	0.724 **	0.616 **	0.698 **	0.634 **	-

Notes: entries above the diagonal concern Spearman correlations; entries below the diagonal concern Pearson correlations. * *p* < 0.05, ** *p* < 0.001.

**Table 3:** Fit indices pertaining to the confirmatory factor analytic models.

Model	*n*	*X* ^2^	df	RMSEA	CFI	TLI	SRMR	RMSEA (IC 90%)
Four-factor CFA	212	496.317	224	0.076	0.977	0.974	0.065	0.076 (0.067–0.085)
Two-factor CFA	212	573.119	229	0.084	0.970	0.967	0.072	0.084 (0.076–0.093)

Notes: CFA, confirmatory factor analysis; df, degrees of freedom; RMSEA, root mean square error of approximation; CFI, comparative fit index; TLI, Tucker–Lewis index; SRMR, standardized root mean squared residual.

**Table 4:** Correlations for age, years of service, ERCBS-T and ERCBS subscales.

	1	2	3	4	5	6	7
1. Age	-	0.626 **	−0.215 **	−0.127	−0.087	−0.220 **	−0.265 **
2. Years of service	0.620 **	-	−0.167 *	−0.104	−0.067	−0.174 *	−0.240 **
3. ERCBS-T	−0.207 **	−0.189 **	-	0.887 **	0.811 **	0.631 **	0.767 **
4. Depression–Anger	−0.132 *	−0.138 *	0.895 **	-	0.880 **	0.321 **	0.457 **
5. Fear–Anxiety	−0.084	−0.082	0.836 **	0.881 **	-	0.228 **	0.375 **
6. Confident–Relaxed	−0.224 **	−0.185 **	0.642 **	0.296 **	0.218 **	-	0.805 **
7. Cheerful–Excited	−0.265 **	−0.229 **	0.760 **	0.437 **	0.381 **	0.797 **	-

Notes: entries above the diagonal concern Spearman correlations; entries below the diagonal concern Pearson correlations. ERCS subscales: Depression–Anger, Fear–Anxiety, Confident–Relaxed, Cheerful–Excited. * *p* < 0.05; ** *p* < 0.01.

## Data Availability

The data presented in this study are available on request from the corresponding author.
